# Identification of runs of homozygosity affecting female fertility and milk production traits in Finnish Ayrshire cattle

**DOI:** 10.1038/s41598-020-60830-9

**Published:** 2020-03-02

**Authors:** K. Martikainen, M. Koivula, P. Uimari

**Affiliations:** 1 Department of Agricultural Sciences, University of Helsinki, P.O. Box 28, FI-00014 Helsinki, Finland; 2 Natural Resources Institute Finland (Luke), Green Technology, FI-31600 Jokioinen, Finland

**Keywords:** Animal breeding, Inbreeding

## Abstract

Inbreeding gives rise to continuous lengths of homozygous genotypes called runs of homozygosity (ROH) that occur when identical haplotypes are inherited from both parents. ROHs are enriched for deleterious recessive alleles and can therefore be linked to inbreeding depression, defined as decreased phenotypic performance of the animals. However, not all ROHs within a region are expected to have harmful effects on the trait of interest. We aimed to identify ROHs that unfavourably affect female fertility and milk production traits in the Finnish Ayrshire population. The estimated effect of ROHs with the highest statistical significance varied between parities from 9 to 17 days longer intervals from calving to first insemination, from 13 to 38 days longer intervals from first to last insemination and from 0.3 to 1.0 more insemination per conception. Similarly, for milk production traits ROHs were associated with a reduction of 208 kg for milk yield, 7 kg for protein yield and 16 kg for fat yield. We also found regions where ROHs displayed unfavourable effects across multiple traits. Our findings can be exploited for more efficient control of inbreeding depression, for example by minimizing the occurrence of unfavourable haplotypes as homozygous state in breeding programmes.

## Introduction

In dairy cattle breeding, genetic gain of economically important traits has been achieved by intensive selection. The downside of intensive selection is, however, increased levels of inbreeding and, thus, possible accumulation of recessive deleterious alleles. This, in turn, results in inbreeding depression, which is defined as a reduction in average phenotypic performance^1^. Managing inbreeding is crucial to maintaining the profitability of dairy cattle production, since inbreeding depression has been linked with impairment in economically important traits such as fertility and milk production^2–5^.

With the availability of single-nucleotide polymorphism (SNP) marker panels, the effects of inbreeding can be investigated at the genomic level. Inbreeding gives rise to continuous segments of homozygous genotypes known as runs of homozygosity (ROH), which are present in an individual if both parents transmit identical haplotypes to their offspring^6^. Since ROHs unlikely arise by chance, they are considered good estimates of inbreeding^7^. Inbreeding depression is assumed to be mostly caused by homozygosity for deleterious recessive mutations, which occur typically at low frequency in a population^8^. ROHs are enriched for these deleterious recessive alleles and can, therefore, be linked to inbreeding depression^9^. Previous research has found an association between increased inbreeding based on ROH (F_ROH_) and reduced fertility or milk production. For example, a 1% increase in F_ROH_ was associated with 1.72 days increase in days open and 20 kg decrease in 205-day milk yield^3^, and also with 0.4 days longer insemination interval in heifers^5^.

Since the patterns of inbreeding vary throughout the genome^10^, identification of genomic regions with associations between ROHs and impaired fertility or milk production traits will allow breeding programmes to balance more efficiently between genetic gain and levels of inbreeding. Pryce *et al*.^4^, Kim *et al*.^11^ and Martikainen *et al*.^12^ reported unfavourable phenotypic effects on reproduction traits for several genomic regions in an ROH in Holstein, Jersey and Finnish Ayrshire cattle, respectively. Pryce *et al*.^4^ also found an association between several genomic regions in ROH and decreased milk yield in Holstein and Jersey cattle. However, these studies did not account for the effect of different ROH genotypes on the regions of interest. As noted by Howard *et al*.^13^, within a region, the majority of ROH genotypes may have neutral or even favourable effects on the phenotype, thus hiding the effect of infrequent unfavourable ROH genotypes. Howard *et al*.^13^ presented a method to estimate the effect separately for each unique ROH genotype. Using this method, Baes *et al*.^14^ were able to identify genotypes within ROHs having unfavourable effects on production, reproduction and health traits in Canadian Dairy cows. For example, the ROH genotype with the most extreme effect was estimated to increase the number of services by 0.5 for heifers (chromosome 16) and by 0.6 for cows (chromosome 11). For the 305-day milk yield, the animals with the most extreme ROH genotype on chromosome 11 were estimated to produce on average 690 kg less milk than animals without ROH.

The aim of our study was to identify ROH genotypes with unfavourable effects on female fertility traits and milk production traits in the Finnish Ayrshire population. We estimated the difference in phenotype between cows with the ROH genotype and cows without ROH. In addition, we identified regions harbouring multiple unfavourable ROH genotypes as well as ROH genotypes affecting multiple traits.

## Results

In this study, we identified the ROH genotypes having an unfavourable effect on female fertility and milk production traits in Finnish Ayrshire cattle. The fertility traits included interval from calving to first insemination (ICF), interval from first to last insemination (IFL) and number of inseminations (AIS). The fertility traits were considered separately for heifers (0), for first parity cows (1), for second parity cows (2) and for third parity cows (3). The milk production traits included deregressed proofs (DRP) of the first lactation of milk yield (MILK), protein yield (PROT) and fat yield (FAT).

**Figure 1:**
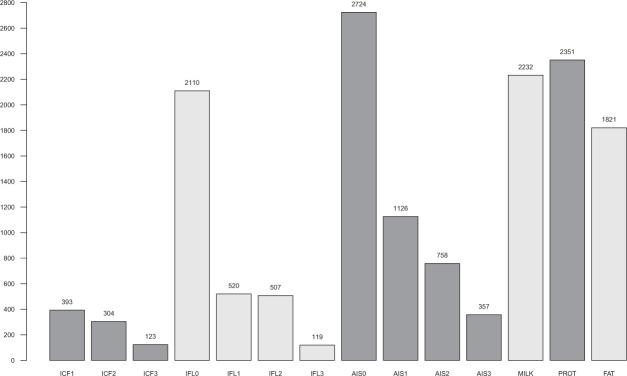
Total number of unfavourable genotypes within the runs of homozygosity (ROH) with P-values < 0.05 and frequencies at least 0.01 for heifers (0) and parities 1, 2 and 3 for the interval from calving to first insemination (ICF), interval from first to last insemination (IFL) and number of inseminations (AIS), and for milk yield (MILK), protein yield (PROT) and fat yield (FAT).

The total number of unfavourable genotypes associated with the fertility traits exceeding the t-statistics cutoff value of 2.326 varied from 2,724 (AIS0) to 119 (IFL3) (Fig. 1). Most of the genotypes that exceeded the cutoff value were associated with the heifer traits and the least for the third-parity cow traits, which was expected considering the larger number of animals with both genotypic and phenotypic records for heifers than for the third-parity cows. For the milk production traits, the total number of unfavourable genotypes exceeding the t-statistics cutoff value was 2,232 for MILK, 2,351 for PROT and 1,821 for FAT (Fig. 1).

### Unfavourable genotypes associated with fertility

We identified genotypes within ROHs with significant unfavourable effects on each of the fertility traits examined. The most significant genotypes for each parity of each trait with the estimated effect of genotypes are presented in Table 1. Animals with the most significant unfavourable genotype showed 9.2, 9.5 and 17.1 days longer ICF for first-, second- and third-parity cows, respectively (the corresponding P-values were 2.22e-04, 1.92e-04 and 2.20e-04, respectively). Similarly, animals with the most significant unfavourable tested genotype| showed 12.8, 13.2, 29.6 and 37.7 days longer IFLs for heifers, first-, second- and third-parity cows, respectively (the corresponding P-values were 4.8e-08, 2.9e-05, 5.0e-06 and 1.2e-04, respectively). For AIS, the estimated effects of the most significant unfavourable genotypes were 0.26, 0.48, 0.58 and 0.95 more inseminations for heifers, first-, second- and third-parity cows, respectively (the corresponding P-values were 8.6-e07, 2.3-e06, 1.8e-05 and 2.3e-05, respectively).

**Table 1:** The most significant unfavourable genotypes within the runs of homozygosity (ROH) for heifers (0) and parities 1, 2 and 3 for the interval from calving to first insemination (ICF), interval from first to last insemination (IFL) and number of inseminations (AIS).

Trait	CHR	Position (Mb)	Length (Mb)	Frequency	Median length of full ROH (Mb)	b (SE)	P-value
ICF1	12	65.13–66.37	1.24	0.010	6.76	9.2 (2.5)	2.22e-04
ICF2	10	62.34–64.20	1.86	0.017	7.48	9.5 (2.5)	1.92e-04
ICF3	12	61.67–62.73	1.06	0.018	7.29	17.1 (4.6)	2.20e-04
IFL0	17	9.21–10.31	1.10	0.015	7.98	12.8 (2.4)	4.84e-08
IFL1	5	27.54–31.16	3.62	0.032	10.71	13.2 (3.2)	2.85e-05
IFL2	8	65.01–69.12	4.11	0.015	11.90	29.6 (6.5)	4.96e-06
IFL3	18	8.01–8.85	0.84	0.021	4.83	37.7 (9.8)	1.16e-04
AIS0	17	9.47–10.59	1.12	0.018	3.15	0.26 (0.05)	8.59e-07
AIS1	14	81.76–83.07	1.31	0.010	5.39	0.48 (0.10)	2.26e-06
AIS2	15	7.31–9.15	1.84	0.010	3.33	0.58 (0.14)	1.83e-05
AIS3	18	4.90–6.93	2.03	0.018	4.55	0.95 (0.22)	2.34e-05

Estimate b represents the difference between animals with the ROH genotype and animals without ROH (in days for IFL and ICF, in numbers of inseminations for AIS).

Table 1 also presents the frequencies and lengths of the most significant ROH genotypes and the median length of the full ROH segments across all individuals with the genotype. All of the genotypes were rarely observed; the frequencies of the ROH genotypes (genotype frequency) varied from 0.01 to 0.03. The lengths of the genotypes varied from 0.84 Mb (IFL3) to 4.11 Mb (IFL2) and the median lengths of the full ROH segments, including the unfavourable genotype, varied from 3.15 Mb (AIS0) to 11.90 Mb (IFL2).

Table 2 presents the genomic regions containing genotypes (within the ROHs) that unfavourably affected the fertility traits. Due to the large number of tests, only genotypes with P-values smaller than 1.0e-05 are presented. For IFL0, three regions on chromosomes BTA17 (position 8.63–10.59 Mb), BTA22 (position 6.45–8.06 Mb) and BTA24 (position 58.64–60.58 Mb) were associated with impaired fertility (P-value < 1.0e-05). The estimated effects of the unfavourable genotypes varied from 6.0 days to 12.8 days longer insemination intervals than in animals without an ROH in that region. For IFL2, one genotype on chromosome BTA8 (position 65.01–69.12 Mb) showed an estimated effect of 29.6 days. Five regions on chromosomes BTA2 (position 26.21–27.18 Mb), BTA6 (position 36.81–39.53 Mb), BTA17 (position 9.21–10.59 Mb), BTA19 (position 30.83–31.64 Mb) and BTA22 (position 6.68–10.68 Mb) were associated with the effects of genotypes on AIS0. Animals that had ROH genotypes on any of the five regions had 0.12–0.31 more inseminations than animals not having an ROH in that region. For AIS1, seven genotypes in the same region on chromosome BTA14 (position 81.61–83.87 Mb) showed the estimated effect, varying from 0.45 to 0.48 inseminations.

**Table 2:** Regions with unfavourable genotypes within the runs of homozygosity (ROH) with P-values < 1.0e-05 for heifers (0) and parities 1 and 2 for interval from first to last insemination (IFL) and number of inseminations (AIS).

Trait	CHR	Number of ROH genotypes	Position (Mb)	b	P-value	Frequency of ROH genotypes (min/max)
IFL0	17	5	8.63–10.59	10.5–12.8	4.84e-08	0.01/0.02
IFL0	22	1	6.45–8.06	12.3	9.83e-06	0.01
IFL0	24	9	58.64–60.58	6.0–6.8	6.28e-08	0.05/0.06
IFL2	8	1	65.01–69.12	29.6	4.96e-06	0.01
AIS0	2	1	26.21–27.18	0.22	8.59e-06	0.02
AIS0	6	2	36.81–39.53	0.12–0.16	3.78e-06	0.04/0.09
AIS0	17	2	9.21–10.59	0.26–0.28	8.59e-07	0.01/0.02
AIS0	19	1	30.83–31.64	0.25	8.86e-06	0.02
AIS0	22	8	6.68–10.68	0.28–0.31	3.95e-06	0.01/0.01
AIS1	14	7	81.61–83.87	0.45–0.48	2.26e-06	0.01/0.01

Estimate b represents the difference between animals with the ROH genotype and animals without ROH (in days for IFL and in numbers of inseminations for AIS), while the P-value represents the smallest P-value for the region.

### Unfavourable genotypes associated with milk production traits

We found several ROH genotypes with significant unfavourable associations with milk production traits. The most significant genotypes are presented in Table 3. The estimated unfavourable effects of the most significant genotypes were on average 208 kg for MILK, 7 kg for PROT and 16 kg for FAT (the corresponding P-values were 9.30e-08, 3.60e-10 and 8.60e-09, respectively). The frequencies of the most significant genotypes varied from 0.01 for FAT to 0.06 for PROT. The length of the genotypes varied from 1.08 Mb (FAT) to 1.98 Mb (PROT). The median length of the full ROH segments varied from 7.36 Mb (MILK) to 13.28 Mb (FAT).

**Table 3:** The most significant unfavourable genotypes within the runs of homozygosity (ROH) for milk, protein and fat yield (MILK, PROT and FAT, respectively).

Trait	CHR	Position (Mb)	Length (Mb)	Frequency	Median length of full ROH (Mb)	b in kg (SE)	P-value
MILK	20	31.55–33.15	1.60	0.049	7.36	−207.843 (38.9)	9.30e-08
PROT	13	53.66–55.64	1.98	0.056	12.75	−7.04 (1.12)	3.60e-10
FAT	5	87.02–88.11	1.08	0.012	13.28	−15.68 (1.69)	8.60e-09

Estimate b represents the difference between animals with the ROH genotype and animals without ROH.

Table 4 presents genomic regions containing genotypes (within the ROHs) with unfavourable effects on milk production traits. The same P-value threshold 1.0e-05 was applied as for the fertility traits. Nine regions on eight chromosomes were associated with the effects of genotypes on reduced MILK. These regions were located on chromosomes BTA2 (position 80.54–84.81 Mb), BTA3 (position 52.83–56.18 Mb), BTA5 (positions 89.16–90.75 Mb and 96.55–102.05 Mb), BTA6 (position 115.84–117.01 Mb), BTA13 (position 44.37–58.55 Mb), BTA16 (position 42.21–44.99 Mb), BTA20 (position 29.23–33.31 Mb) and BTA22 (position 58.68–59.46 Mb). The estimated decrease in MILK varied from 141.9 kg to 354.2 kg.

**Table 4:** Regions with unfavourable genotypes within the runs of homozygosity (ROH) with P-values < 1.0e-05 for milk, protein and fat yield (MILK, PROT and FAT, respectively).

Trait	CHR	Number of ROH genotypes	Position (Mb)	b in kg	P-value	Frequency of ROH genotypes (min/max)
MILK	2	5	80.54–84.81	−269.3–−279.5	4.35e-06	0.02/0.02
MILK	3	3	52.83–56.18	−321.6–−329.0	3.11e-06	0.01/0.01
MILK	5	1	89.16–90.75	−218.0	6.36e-06	0.03
MILK	5	1	96.55–102.05	−329.9	6.29e-06	0.01
MILK	6	1	115.84–117.01	−354.2	5.19e-06	0.01
MILK	13	131	44.37–58.55	−145.52–−252.0	1.23e-07	0.03/0.08
MILK	16	2	42.21–44.99	−196.7–−215.9	4.45e-06	0.03/0.04
MILK	20	22	29.23–33.31	−141.9–−217.7	9.30e-08	0.04/0.07
MILK	22	1	58.68–59.46	−341.6	2.99e-06	0.01
PROT	3	1	1.55–2.91	−6.94	4.93e-06	0.03
PROT	3	5	52.83–56.18	−9.7–−11.7	1.32e-07	0.01/0.01
PROT	6	12	72.53–80.66	−6.7–−11.6	2.60e-07	0.01/0.03
PROT	6	1	93.08–94.46	−10.47	8.91e-06	0.01
PROT	8	3	79.50–81.57	−9.8–−10.0	3.51e-06	0.01/0.01
PROT	13	176	42.93–58.55	−4.7–−8.8	3.60e-10	0.02/0.08
PROT	14	11	68.03–73.41	−5.6–−6.6	3.00e-06	0.03/0.04
PROT	16	1	43.64–46.51	−11.1	7.43e-06	0.01
PROT	16	1	52.51–53.92	−9.7	8.71e-06	0.01
PROT	22	1	57.99–59.33	−10.9	7.17e-06	0.01
FAT	5	1	87.02–88.11	−15.7	8.60e-09	0.01
FAT	5	6	109.93–118.35	−7.0–−12.8	1.56e-06	0.01/0.04
FAT	8	46	11.74–20.81	−10.5–−14.8	6.34e-08	0.01/0.02
FAT	13	1	57.32–58.55	−6.6	8.55e-06	0.04
FAT	17	2	7.14–8.03	−11.4–−11.5	2.69e-06	0.01/0.01
FAT	29	5	36.76–37.96	−3.9–−4.5	5.41e-07	0.12/0.14

Estimate b represents the difference between animals with the ROH genotype and animals without the ROH, while the P-value represents the smallest P-value for the region.

Ten regions on seven chromosomes were associated with the effects of genotypes on reduced PROT. The corresponding regions located on chromosomes BTA3 (positions 1.55–2.91 Mb and 52.83–56.18 Mb), BTA6 (positions 72.53–80.66 Mb and 93.08–94.46 Mb), BTA8 (position 79.50–81.57 Mb), BTA13 (position 42.93–58.55 Mb), BTA14 (position 68.03–73.41 Mb), BTA16 (positions 43.64–46.51 Mb and 52.51–53.92 Mb) and BTA22 (position 57.99–59.33 Mb). The estimated unfavourable effect of these genotypes varied from 4.7 kg to 11.7 kg.

Six regions on five chromosomes were associated with the effects of genotypes on reduced FAT. These genotypes were located on chromosomes BTA5 (positions 87.02–88.11 Mb and 109.93–118.35 Mb), BTA8 (position 11.74–20.81 Mb), BTA13 (position 57.32–58.55 Mb), BTA17 (position 7.14–8.03 Mb) and BTA29 (position 36.76–37.96 Mb). The estimated decreasing effect of these genotypes on FAT varied from 3.9 kg to 15.7 kg.

### Shared genotypes between traits

Table 5 presents the unfavourable genotypes identified across traits by accepting only genotypes with P-values < 1.0e-05 on both of the traits examined. Shared genotypes were found only between fertility traits or between milk production traits, and none were found between fertility and milk production traits. For the fertility traits, two genotypes were found on chromosome BTA17 (position 9.21–10.59 Mb) with an increasing effect on both IFL0 and AIS0. These two genotypes were estimated to lengthen the insemination interval from 10.5 days to 12.6 days while increasing the number of inseminations by approximately 0.3 for heifers.

**Table 5:** Regions containing shared unfavourable genotypes within the runs of homozygosity (ROH) between traits (interval from first to last insemination for heifers (IFL0), number of inseminations for heifers (AIS0), milk yield (MILK), protein yield (PROT) and fat yield (FAT) with P-values < 1.0e-05.

Trait 1	Trait 2	CHR	Number of ROH genotypes	Position (Mb)	b on trait 1 (P-value)	b on trait 2 (P-value)
IFL0	AIS0	17	2	9.21–10.59	10.5–12.8 (4.84e-08)	0.26–0.28 (8.59e-07)
MILK	PROT	3	3	52.83–56.18	−321.6–−329.0 (3.11e-06)	−11.3–−11.7 (1.32e-07)
MILK	PROT	13	83	44.37–58.55	−145.5–−252.0 (1.23e-07)	−4.9–−8.8 (3.60e-10)
MILK	FAT	13	1	57.32–58.55	−207.3 (5.31e-07)	−6.6 (8.55e-06)
PROT	FAT	13	1	57.32–58.55	−7.6 (5.80e-09)	−6.6 (8.55e-06)

Estimate b represents the difference between animals with the ROH genotype and animals without ROH (in days for IFL, in numbers of inseminations for AIS and in kg for MILK, PROT and FAT), while the P-value represents the smallest P-value for the region.

Several shared genotypes were found between the milk production traits. Three genotypes on chromosome BTA3 (position 52.83–56.18 Mb) negatively affected both MILK (322–329 kg) and PROT (11–12 kg). On chromosome BTA13, a region at position 44.37–58.55 Mb contained 83 genotypes with decreasing effects on both MILK and PROT. The estimated effect of these genotypes varied from 146 kg to 252 kg less MILK and 5 kg to 9 kg less PROT in contrast to animals without ROHs. These regions also contained one genotype at position 57.32–58.55 with a decreasing effect of 6.6 kg on FAT.

## Discussion

The aim of this study was to identify genotypes in ROHs with unfavourable effects on fertility and milk production traits in Finnish Ayrshire cattle. The total number of unfavourable genotypes (with P-values < 0.05) detected in this study was 15,445, and it varied between traits from 119 for the IFL in third-parity cows (IFL3) to 2,724 for the AIS of heifers (AIS0). However, as reported by Howard *et al*.^13^, the algorithm of the Unfavorable Haplotype Finder software may result in multiple genotypes that seem different at the individual level, but in reality are tagging the same observed ROH. Therefore, the total number of unfavourable genotypes identified by the software was expected to be much larger than the number of genotypes within ROHs with unfavourable effects on the traits examined. For this reason, and due to the tendency of multiple testing to produce false discoveries, we reported only the most significant genotypes for each trait and the regions containing genotypes with P-values smaller than 1.0e-05. Even though these selections may have omitted some genotypes, which would indeed have had a detrimental effect on the traits examined, it was expected to pinpoint those regions in the genome of the Finnish Ayrshires where homozygosity has the most harmful effects population-wise.

The average length of the most significant unfavourable genotypes across traits was 1.8 Mb, while the median length of the full ROH segments was much longer, varying from 3.15 Mb to 13.28 Mb (Table 1). Our results were based on medium-density SNP data that could have caused overestimation of the number of short ROH segments, which in reality are not truly identical by descent^15^. The Unfavorable Haplotype Finder software did not allow for setting a minimum length for ROHs, but we attempted to minimize the occurrence of short nonautozygous ROHs by pruning out SNPs with very low minor allele frequency (MAF) and high linkage disequilibrium (LD), as suggested by Howrigan *et al*.^16^. Howard *et al*.^13^ reported that the size of the full ROH segments can vary among animals, due to recombination occurring at different locations in the animals. The algorithm of the Unfavorable Haplotype Finder software finds the core genotype common to all animals to serve as a tag for the full ROH, and the length of the tag genotype is presumably shorter than that of the full ROH. Since the genotypes identified in this study tagged much longer ROHs, they should not be considered as exact locations in the genome harbouring detrimental quantitative trait loci, but rather as indicators for regions associated with inbreeding depression.

The estimated effect of the most significant genotypes presents the difference in the phenotypic mean between animals with the genotype in ROHs and those without. As noted by Howard *et al*.^13^, comparing animals without ROHs to those having ROHs aligns with the partial dominance hypothesis. This hypothesis suggests that inbreeding depression is due to increased frequency of homozygous deleterious recessive or partially recessive alleles, and it is considered as the main reason for inbreeding depression^17^.

For the ICF, cows of first and second parity with the unfavourable genotype were estimated to have about 9 days longer intervals than cows without the genotype. Previous studies performed with the same software estimated the effect of the most extreme genotype instead of the most significant genotype, yet the results of our study were consistent with Marras *et al*.^18^, who found an effect of 10.5 days for first-parity cows in a Canadian Holstein population and Baes *et al*.^14^, who found an effect of 9.3 days in a Canadian dairy cattle population. For third-parity cows with the most significant genotype, the estimated effects in our study were greater than in previous studies for all fertility traits. However, these previous studies did not estimate the effects separately for cows of different parities, which complicates the comparison of results. For the IFL, the effect was about 13 days for heifers and from 13 to 38 days for cows. Baes *et al*.^14^ and Marras *et al*.^18^ studied the interval from first insemination to calving, which is defined as the IFL plus the gestation length. However, Pereira *et al*.^19^ showed that increased inbreeding did not affect the gestation length, suggesting why the lengthening in first insemination to calving can be compared with that in IFL caused by the unfavourable genotype. Marras *et al*.^18^ found an effect of around 14 days for heifers and 23 days for first-parity cows. Corresponding results from the study by Baes *et al*.^14^ included 17 days for heifers and 18 days for cows. The effect of the most significant genotype for the number of inseminations (AIS) was about 0.3 more inseminations for heifers and from 0.5 to 1.0 more insemination for cows. The corresponding results from Marras *et al*.^18^ were about 0.6 more inseminations for both heifers and first-parity cows, while the results from Baes *et al*.^14^ were about 0.5 more inseminations for heifers and 0.6 more for cows.

The effects of the most significant unfavourable genotypes affecting the milk production traits identified in this study were smaller than those reported in previous studies. Our estimates of the unfavourable effects included a reduction of 210 kg in MILK, 7 kg in PROT and 16 kg in FAT, while Marras *et al*.^18^ obtained a reduction of about 680 kg in 305-day milk yield, 22 kg in 305-day protein yield and 29 kg in 305-day fat yield, and Baes *et al*.^14^ a reduction of about 920 kg in 305-day milk yield, 29 kg in 305-day protein yield and 32 kg in 305-day fat yield. Even considering the most extreme genotypes instead of the most significant genotypes found in our study, the effects were about 350 kg less MILK, 13 kg less PROT and 16 kg less FAT than animals without ROH genotypes. Overall, the effects of the most significant genotypes were strongly unfavourable for each of the fertility and production traits examined, which indicates that controlling the homozygosity of these and other unfavourable genotypes can be beneficial in breeding programmes.

We found several regions containing genotypes that were associated with impaired fertility and milk production, with P-values smaller than 1.0e-05. If the P-values were adjusted for the number of tests (P < 0.05/15,445), where 15,445 is the total number of unfavourable genotypes of all traits detected using the software, the threshold would have been about 3.0e-06. However, a less stringent P-value threshold of 1.0e-05 was selected, because Bonferroni correction assumes independent testing, but both fertility and milk production traits are correlated between parities, between ICF, IFL, and AIS and between MILK, PROT and FAT. The number of unfavourable genotypes identified from these regions varied from 1 to 9 for fertility and from 1 to 176 for milk production traits. Although many of these genotypes may have been tagging the same unfavourable ROH, we expected that when the number of the genotypes increased, the number of the various ROH genotypes with an unfavourable effect on the trait of interest would also increase.

The previous study by Martikainen *et al*.^12^ used the same data to identify genomic regions where an increased ROH-based inbreeding coefficient was associated with impaired IFL. In Martikainen *et al*.^12^ different ROH genotypes were not considered, and the regions identified on chromosomes 2, 18 and 22 (IFL0) and on chromosome 15 (IFL2) differed from those identified in the present study. However, all the regions found in the previous study by Martikainen *et al*.^12^ contained several unfavourable genotypes that were also detected by us here, but their statistical significance was lower than the threshold used in Table 2. The proportion of ROHs with neutral or favourable effects on these regions was presumably low, suggesting these regions showed inbreeding depression even when all the various ROH genotypes were tested together. On the other hand, the regions found in the present study that were not revealed in the previous study by Martikainen *et al*.^12^ implies that these regions also contained ROHs that were neutral or even positively associated with IFL or AIS. Since selection may have created both detrimental and beneficial homozygosity, the effective control of inbreeding depression requires identification of the carriers of the unfavourable genotypes instead of simply avoiding the mating of any ROH carriers.

Nine regions on chromosomes 2, 3, 5, 6, 13, 16, 20 and 22 carried unfavourable genotypes for MILK, as did 10 regions on chromosomes 3, 6, 8, 13, 14, 16 and 22 for PROT and six regions on chromosomes 5, 8, 13, 17 and 29 for FAT. The estimated effect of the unfavourable genotypes in these regions varied from 140 to 350 kg for MILK, from 5 to 12 kg for PROT and from 4 to 16 kg for FAT. Most of these regions contained only a few unfavourable genotypes, which were in reality expected to tag the same ROHs. However, two regions were identified from chromosomes 8 and 13 with dozens of genotypes. A region on chromosome 8 contained 46 genotypes associated with reduced FAT, and a region on chromosome 13 contained 131 genotypes associated with reduced MILK and 176 genotypes associated with reduced PROT. The large number of genotypes indicates that they were tagging many different ROH genotypes, suggesting that inbreeding is generally harmful in these regions. The same region on chromosome 13 was also identified when scanning genotypes that showed unfavourable effects across multiple production traits. We identified 83 common genotypes between MILK and PROT on chromosome 13. One of these genotypes was common between all three milk production traits. The detrimental effects of homozygosity in this region may have been due to the guanine nucleotide-binding protein, alpha-stimulating (GNAS) locus, which has a highly significant (P-value 2.18e-19 or smaller) association with milk, protein and fat yields^20^. Three genotypes on chromosome 3 were also common between MILK and PROT, as were two genotypes from chromosome 17 common between IFL0 and AIS0. However, it should be noted that in this study only the shared identical genotypes with P-values < 1.0e-05 for both traits were considered. As pointed out by Howard *et al*.^13^, the regions with unfavourable effects across multiple traits may be particularly interesting, because they are sensitive to inbreeding and thus strongly reduce the phenotypic performance of an individual. Moreover, Howard *et al*.^13^ reported that some long ROH may be locally unfavourable around the identified genotype, but have an overall favourable effect on some trait with an economical importance. Therefore, more research is needed to analyse if the unfavourable ROHs identified in this study have positive or negative effects on other traits under selection. In addition, given the large number of unfavourable ROHs, an optimal use of these ROHs in breeding programmes should be investigated; a possible approach could be e.g. use of inbreeding load matrix for mating decision to minimize the occurrence of the unfavourable haplotypes as a homozygous state^13,21^.

## Conclusions

Identification of unfavourable genotypes within ROHs revealed several regions from the genome of the Finnish Ayrshire cattle showed inbreeding depression of female fertility and milk production traits. These regions have previously remained undetected when the same data were used, but all ROH genotypes were considered to have a negative effect on the traits examined. The results of this study support the hypothesis that not all ROH genotypes in a region have unfavourable effects. Therefore, different ROH genotypes should be tested individually for more precise detection of genomic regions associated with impaired phenotypic performance. The results of this study can be used to investigate the effects of the genes in these regions, or in the breeding programme to more efficiently control inbreeding depression.

## Materials and Methods

### Genotypic data

No animal experiments were carried out for this study, and therefore ethical approval was not required. The genotypic data used in this study were the same as in Martikainen *et al*.^5^ and were obtained from the Nordic Cattle Genetic Evaluation (NAV) (Aarhus, Denmark). The original genotypes of 19,075 Finnish Ayrshire females were derived from the Illumina BovineLD v.2 BeadChip low-density panel^22^, which contains 7,931 SNPs. Prior to the analyses, the genotypes were imputed to 50 K density. Imputation was performed with Fimpute software^23^, using default parameters. The reference population comprised Nordic Red AI bulls with 50 K genotypes.

The SNP positions were based on a UMD 3.1 assembly. Quality control of the imputed genotypes consisted of removing the SNPs with MAF less than 0.01. No filtering based on the deviation from the Hardy-Weinberg equilibrium was performed, since inbreeding is one possible cause for the deviation. High LD increases the probability of detecting nonautozygous ROHs that are formed by chance^16^. Therefore, the SNPs were pruned for LD, using the *–indep* command in PLINK version 1.9^24,25^, which removed the SNP that had the variance inflation factor greater than 10 (corresponding to r^2^ > 0.9) within a 50-SNP window. After quality control and LD-pruning, 29,227 SNPs were available for analysis.

### Phenotypes of female fertility

Three female fertility traits including interval (in days) from calving to first insemination (ICF), interval (in days) from first to last insemination (IFL), and a number of inseminations (AIS) were used in the analysis. The IFL and AIS were considered separately for heifers (parity 0) and for cows of first, second and third parity (parities 1, 2 and 3). The ICF was considered separately for cows with parities 1, 2 and 3. The raw phenotypic values were adjusted for the main systematic effects, including age at first insemination, herd-birth year or herd-year of first calving (for heifers and cows, respectively), insemination year-month (IFL and AIS) and calving year-month (ICF). Descriptive statistics of adjusted observations for each trait at each parity are presented in Table 6. The phenotypic records and solutions for the systematic effects of the fertility traits were derived from NAV and Faba (The Finnish Animal Breeding Association, Hollola, Finland). The combined fertility and genotypic data included observations from 13,712 animals.

**Table 6:** Descriptive statistics of fertility traits adjusted for the main systematic effects for the interval from calving to first insemination (ICF), interval from first to last insemination (IFL) and number of inseminations (AIS), and deregressed proofs of the first lactation of milk yield (MILK), protein yield (PROT), and fat yield (FAT).

Trait	Number of animals	Mean	SD
ICF1	9 453	0.25	23.89
ICF2	5 067	−0.13	24.08
ICF3	1 509	−0.96	22.51
IFL0	12 878	1.65	37.54
IFL1	9 546	3.72	52.48
IFL2	5 131	5.14	51.55
IFL3	1 567	6.47	49.12
AIS0	13 261	0.07	0.99
AIS1	9 323	0.15	1.13
AIS2	4 918	0.22	1.13
AIS3	1 453	0.22	1.08
MILK	12 233	270.11	1112.51
PROT	12 233	5.40	35.43
FAT	12 233	2.81	41.30

ICF, IFL and AIS are considered for heifers (0) and for parities 1, 2 and 3.

### Phenotypes of milk production traits

The milk production traits used in the analysis included deregressed proofs (DRPs) of the first lactation of milk yield (MILK), protein yield (PROT), and fat yield (FAT) expressed in kilograms. For the calculation of DRPs, the estimated breeding values (EBVs) of milk, protein, and fat yield were derived from the routine NAV milk production test day data. First, the effective record contributions (ERCs) were calculated by the ApaX99 program^26^ for all animals in the pedigree. The heritabilities used in the ERC approximations were 0.45, 0.43 and 0.44 for milk, protein, and fat yields, respectively. Next, the DRPs were calculated for all cows, using the EBVs and ERCs. The DRPs were obtained, using the Broyden method in option DeRegress in the MiX99 software^27^. The ERCs were used as weighting factors in the deregression. The three traits were deregressed simultaneously, but assuming zero genetic and residual correlations between the traits. Reliability of each individual DRPi was calculated as r^2^_DRPi_ = ERC_i_/(ERC_i_ + λ), where λ = (1 − h^2^)/h^2^. Only DRPs with reliability r^2^_DRP_ ≥ 40% were accepted as observations for the analysis. All observations with r^2^_DRP_ < 40% for MILK, PROT or FAT were removed from the data. The combined production and genotypic data included observations from 12,233 animals. Descriptive statistics of milk production DRPs are presented in Table 6.

### Identification of unfavourable ROH genotypes

Genotypes within ROHs with unfavourable effect on female fertility or milk production traits were detected, using Unfavorable Haplotype Finder software^13^. A complete description of the algorithm is presented in Howard *et al*.^13^. The algorithm is comprised of three steps. In step 1, ROH genotypes (i.e. identical haplotypes from parents) with no heterozygous SNPs and a frequency above the user-defined threshold are placed in the ROH category, while the other genotypes are placed in the non-ROH category. In this study, the threshold for the frequency of unique ROH genotype was set as 0.01 to exclude rare genotypes. Then, the ROH genotypes associated with unfavourable effects on the phenotype were identified, and a sliding window of decreasing size was used to detect the core ROH genotype serving as a tag for the full ROH. In this study, the window size was decreased by 50 SNPs to 15 SNPs at 5-SNP intervals and the cutoff value for the mean phenotype of the unfavourable genotype was generated by performing 1,000 permutations for random genomic regions for each trait separately. Based on the permutations, a mean phenotypic value with a statistical significance between 0.05 and 0.10 was selected as a cutoff value. Genotypes with mean phenotypic values for ICF, IFL and AIS above the cutoff value, or for MILK, PROT and FAT below the cutoff value, were considered as unfavourable and tested in step 2.

In step 2, the significance of each genotype detected in step 1 was tested, using a linear mixed model. In step 3, the nested genotypes were removed.

The linear mixed model used in step 2 was:

$${y}_{i}={\boldsymbol{X}}b+{\boldsymbol{Z}}a+e,$$

where *y*_*i*_ is a preadjusted phenotype (fertility traits) or DRP (production traits) of the trait of interest, *b* is a vector including the effect of ROH genotype, *a* a vector of random additive genetic effects and *e* a vector of random residual effects. Within the fertility traits (AIS, ICF and IFL), parities 0 (heifers for AIS and IFL), 1, 2 and 3 were analysed separately. The additive genetic effects were assumed to be normally distributed with N(0, **A**σ^2^_a_), where **A** is the pedigree-based additive relationship matrix and σ^2^_a_ the additive genetic variance. The residual effects were assumed to be normally distributed with N(0, **I**σ^2^_e_), where **I** is an identity matrix and σ^2^_e_ the residual variance. The variance components of the fertility traits used in the analysis were the same as in the Nordic fertility evaluation^28^. For the production traits, the variance components were estimated by Average Information Restricted Maximum Likelihood (AI-REML), using the DMU software^29^. For each window and unique ROH genotype, two groups were formed: one including animals with the ROH genotype tested and the other group including animals that did not have an ROH in the window tested. A one-sided t-test was performed between these groups. In step 3, the nested windows were removed. The frequencies of the genotypes detected and the median lengths of the full ROH segments were calculated, using an R^30^ script by Jeremy Howard (J. Howard, Smithfield Premium Genetics, Roanoke Rapids, NC, USA, personal communication).

## Data Availability

The data that support the findings of this study are available from NAV, Aarhus, Denmark and Faba, Hollola, Finland. Restrictions may apply to the availability of these data, which were used under agreement for this study, and so are not publicly available. Data are however available from the corresponding author upon reasonable request and with permission of NAV and Faba.
